# Second Primary Malignancies of the Bones and Joints: More Common than Expected in Osteosarcoma Patients

**DOI:** 10.5435/JAAOSGlobal-D-22-00275

**Published:** 2023-01-24

**Authors:** Isaac G. Freedman, Hollie N. Dowd, Meera M. Dhodapkar, Scott J. Halperin, Jonathan N. Grauer

**Affiliations:** From the Department of Orthopaedics and Rehabilitation, Yale University School of Medicine, New Haven, CT (Dr. Freedman, Ms. Dhodapkar, Mr.Halperin, and Dr. Grauer) and Yale School of Public Health, New Haven, CT (Ms. Dowd).

## Abstract

Osteosarcoma is the most common primary bone tumor in children, adolescents, and young adults. Second primary malignancies (SPMs) are a potential serious long-term event that can occur in osteosarcoma survivors.

**Methods:**

The National Cancer Institute's Surveillance, Epidemiology, and End Results 18 database was queried for all osteosarcoma cases from 2000 through 2015. Standardized incidence ratio (SIR) and absolute excess risk (AER) of SPM per 10,000 persons (AER) relative to representative population-level data were calculated across for various anatomic locations.

**Results:**

In total, 3438 patients with osteosarcoma were identified. Of these patients, 79 (2.3%) developed SPMs, with an SIR of 2.84 (95% confidence interval [CI] 2.35 to 3.39, *P* < 0.0001) and an AER of 44.96. The most common SPMs were tumors of the bones or joints (SIR 73.07, CI, 38.90 to 124.94, *P* < 0.0001, AER 7.48), tumors of soft tissues including the heart (SIR 15.19, CI, 5.58 to 33.07, *P* < 0.0001, AER 3.27), and leukemia (SIR 22.28, CI, 15.03 to 31.80, *P* < 0.0001, AER 16.74).

**Conclusion:**

The overall incidence of SPMs in osteosarcoma survivors was significantly higher than would otherwise be expected for this population. Considering the occurrence and targeting surveillance for SPM in the osteosarcoma patient population is warranted.

Osteosarcoma is the most common primary bone tumor in children, adolescents, and young adults worldwide, with an incidence of 3.4 million people per year.^1^ It is usually found in appendicular skeleton, such as the distal femur (43%), proximal tibia (23%), or humerus (10%), but may also arise axially.^2^ Although most common in younger patients, there is an increasing prevalence among elderly patients.^3,4^

With improvements in treatments leading to greater numbers of osteosarcoma survivors, there is a critical need for more information regarding the potential development of second primary malignancies (SPMs), sometimes also referred to as second malignant neoplasms (SMNs). Treatment-related effects have been implicated in the increased rate of many second cancers, including breast cancer^5^ and Hodgkin lymphoma.^6^ However, there is also evidence from both prospective and retrospective studies that individuals who develop a first primary cancer may inherently be at greater risk of developing subsequent cancers because of complex genetic, environmental, and behavioral factors, which are not fully understood.^7,8^

## Rationale

In the largest and most recent study to date analyzing SPMs in patients with a history of osteosarcoma, a 2014 analysis of the Surveillance, Epidemiology, and End Results (SEER) database from 1973 to 2010 by Lee et al. found the cumulative incidence of SPMs to be 2.1% with a 10-year SPM risk of 4.0% and a 20-year incidence of 7.4%, and an all sites standardized incidence ratio (SIR) of SMNs of 4.7 from 1986 through 2010.^9^ This study was based on only 60 SPMs across nearly 40 years of nationwide data, about half of what is currently in the database. Other estimates of 10-year cumulative incidence of SPMs by various retrospective osteosarcoma studies, including single-institutional studies^10-12^ and international studies of large databases,^13^ have ranged between 2.0% and 4.9%.^10,11,12,13^ Because of the rarity of osteosarcoma and SPMs in patients with osteosarcoma, updates to the estimates provided by Lee et al. including years since 2010 may further inform current clinical practice for osteosarcoma survivors.

The aim of this study was to evaluate whether individuals with osteosarcoma in a more recent study period—here, 2000 through 2015—captured by the large national SEER cancer database were at an increased risk of SPMs and to determine whether SPMs are more common in specific anatomic locations in this population.

## Methods

### Study Design, Setting, and Participants

Patient information was abstracted from the SEER 18 2000 to 2015 database (www.seer.cancer.gov). The study population rate file was “incidence – SEER 18 Regs excluding AK Research Data, November 2017 Sub (2000 to 2015) <Katrina/Rita Population Adjustment>.”

This database uses information from 18 population-based cancer registries in both metropolitan and rural areas across 13 states (Alaska, California, Connecticut, Georgia, Hawaii, Iowa, Kentucky, Louisiana, Michigan, New Jersey, New Mexico, Utah, and Washington), excluding research data from Alaska, and adjusted for changes in reporting in the Louisiana Cancer Registry and the Gulf Coast region affected by hurricanes Katrina and Rita for the second half of 2005. The total study population covers approximately 28% of the US population.

Information on expected incidence was calculated using the referent rate file that corresponded to the population incidence file: “SEER 18 (excl AK) 2000-2015 (Nov 2017 sub), Race (WU/B/O), Event: Site recode B ICD-O-3/WHO 2008.” This file is provided by SEER for producing “All Races Combined” statistics and includes referent rate data.

The SEER program annually collects data regarding patient demographics, cancer incidence, and mortality. The data elements include cancer type, tumor site, tumor morphology, stage at diagnosis, first course of treatment, and follow-up time. Primary neoplasm status for all individuals in the covered population was recorded regularly, typically annually, during the study period. Temporal sequence number for each primary malignancy was recorded in the SEER database and used for this analysis.

Patients were identified for inclusion in the study based on the reported primary malignancy of osteosarcoma. Patients were excluded if their first primary malignancy on record was not osteosarcoma or if their neoplasms were recorded not to exhibit malignant behavior (erroneous data for osteosarcoma). Death-only and autopsy-only cases were also excluded from our analysis because of limited information about those cases.

Primary malignancies were identified using SEER sequence number. The possibility of a malignancy detection bias, where a patient diagnosed with a first primary malignancy may be diagnosed with other primary malignancies immediately afterward because of increased diagnostic scrutiny, was mitigated by excluding patients who were diagnosed with a malignancy within 6 months of their initial osteosarcoma diagnosis. Lymphomas and leukemias were classified as lymphatic and hematopoietic diseases, and all other malignancies were classified as solid tumors.

### Patient Data

For patients identified for inclusion in this study, demographic data were abstracted. These variables included age, sex, and race. Additional data abstracted included years of follow-up, number of SPMs, and latency of SPM development.

Follow-up in the SEER database is based on follow-up protocols that vary across institutions and locations based on hospital admissions and outpatient records and are coordinated by the National Cancer Institute. Typically, follow-up is generated each month based on a control list of patients registered with a neoplasm at an institution. If patients return to the institution, their information is obtained and extracted by the reporting facility; if the patient does not return to the facility, some health information departments will automatically route the patient record to the cancer registry if a cancer diagnosis, current or past, is coded. However, if a patient does not return to a treatment facility and an automatic registry update is not received, follow-up letters are mailed to the managing or referring physician and then to the patient, family members, or contacts if the patient has not been seen by their physician in the past month.^14^

The SEER cancer registries must meet or exceed 95% successful follow-up and is calculated on all eligible patients, both living and dead.^15^ More information on follow-up is available at training.seer.cancer.gov.

Other clinical data such as further clinical cancer characteristics and specific details regarding treatment types and durations were not available in SEER at the time of this analysis. For each patient, it was recorded if, when, and where development of a SPM occurred. Detailed histologic classification and treatment information were not available.

### Statistical Analysis

The SEER database localized SPMs to 119 anatomic site categories. For each of the 119 identified anatomic sites, the following were calculated using a multiple primaries-standardized incidence ratio session of the SEER*stat program (version 8.1.2): total observed SPMs, SIR, 95% confidence interval (CI), *P*-value, and absolute excess risk (AER) per 10,000 person-years.

SIRs were calculated using the SEER*stat MP-SIR procedure, which compares observed SPM rates in the study population with the referent rate file. When not available using SEER*stat, exact *P*-values were calculated using the procedure described in the study of Altman and Bland.^16^ SIRs are defined as the number of observed SPMs divided by the number of expected SPMs in the osteosarcoma population for a given tumor site location. The number of expected malignancies was calculated using the baseline rate of a given malignancy in individuals without osteosarcoma in the SEER 18 sample cohort found in the referent rate file described above.

AER was defined as the number of additional persons diagnosed with a SPM in the osteosarcoma population over the expected number of people in the nonosteosarcoma population in the corresponding referent cohort divided by the total person years at risk and is expressed as a value per 10,000 person-years.

All statistical tests were corrected for multiple comparisons using the Bonferroni procedure. This study was exempted from review by our intuition's institutional review board.

## Results

### Demographics

In total, 3,438 patients with osteosarcoma were identified. Age, sex, and race demographics are presented in Table 1. The median (interquartile range [IQR]) age at diagnosis for the primary malignancy was 21.5 (10.5-54.5) years old. The patient population consisted of 1848 (53.8%) male patients and 1590 (46.2%) female patients. The racial makeup of the group was predominantly White (75.1%).

**Table 1:** Demographics

Variable	Value
Total number of patients, n	3438
Age at diagnosis, median (IQR) years	21.5 (10.5-54.5)
Sex, n (%)	
Male	1848 (53.8)
Female	1590 (46.2)
Race, n (%)	
White	2582 (75.1)
Black	568 (16.5)
Other	288 (8.4)
Total number of SPMs, n (%)	79 (2.3)
Latency of SPM, median (IQR) months	42 (24-104)

IQR = interquartile range, SPM = second primary malignancy

Given that this was representative of an overall population coverage of 106,879,966 individuals in the SEER database, this corresponds to an osteosarcoma prevalence of 3.2 cases per 100,000 individuals.

**Figure 1:**
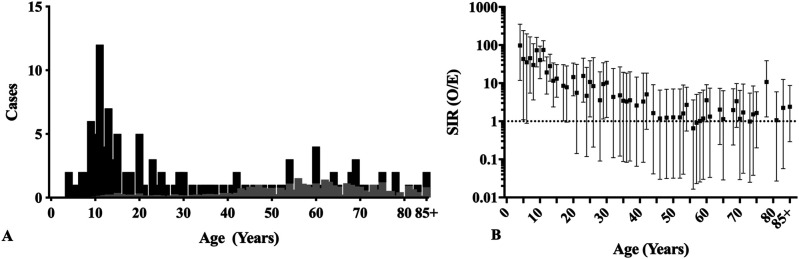
**A**, Chart showing SPM by age at diagnosis: black—observed; gray—expected. **B**, Chart showing SIR by age (95% CI). Figure 1 shows the age that patients developed an SPM of any location at least 6 months after diagnosis of osteosarcoma as a first primary malignancy. SPM = second primary malignancy, SIR = standardized incidence ratio, CI = confidence interval, O/E = observed/expected

SPMs were most common among patients younger than 40 years, with a peak of 12 patients aged 11 years (the mode), Figure 1A. The SIR of SPM was significantly increased in most ages younger than 20 years, with a peak SIR of 97.6 (95% CI, 11.8 to 352.5) for patients aged 4 years (Figure 1B).

### Patients Diagnosed With Osteosarcoma as a First Primary Malignancy Are at Increased Relative and Absolute Risk for Developing Second Primary Malignancies Relative to Representative Control Subjects

**Figure 2:**
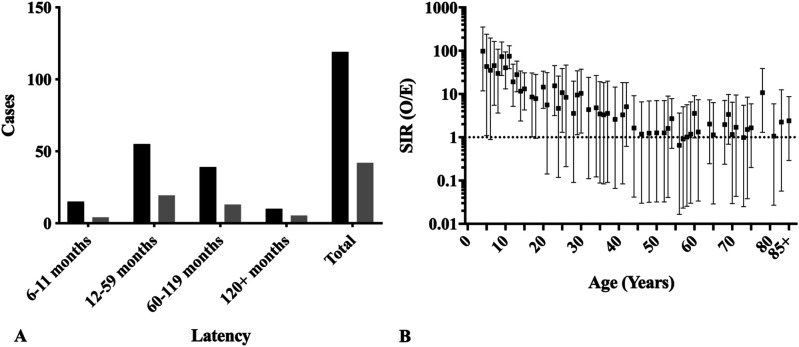
**A**, Chart showing latency to diagnosis of SPM: black—observed; gray—expected. **B**, Chart showing SIR by latency to diagnosis of SPM (95% CI). Figure 2 portrays the latency of development of SPMs. SPM = second primary malignancy, SIR = standardized incidence ratio, CI =confidence interval, O/E = observed/expected.

Of the 3,438 cases of osteosarcoma, 79 (2.3%) patients with SPMs were identified with a median SPM latency (i.e., time to diagnosis of SPM) of 42 months (interquartile range 24-104), Table 1 and Figure 2A. The SIR of SPM across all sites was significant for latency periods from 6 through 11 months (SIR: 4.18, CI, 2.01 to 5.92, *P* < 0.0001), 12 through 59 months (SIR: 2.84, CI, 2.14 to 3.69, *P* < 0.0001), and 60 through 119 months (SIR: 3.01, CI, 2.14 to 4.11, *P* < 0.0001), but not for 120+ months (SIR: 1.84, CI, 0.88 to 3.39, *P* = 0.07), Figure 2B. No individuals in this cohort were diagnosed with more than one SPM.

### Second Primary Malignancies in Patients With Osteosarcoma Are More Likely to Arise From the Bones or Joints Than Are Malignancies in Healthy Control Subjects

**Figure 3:**
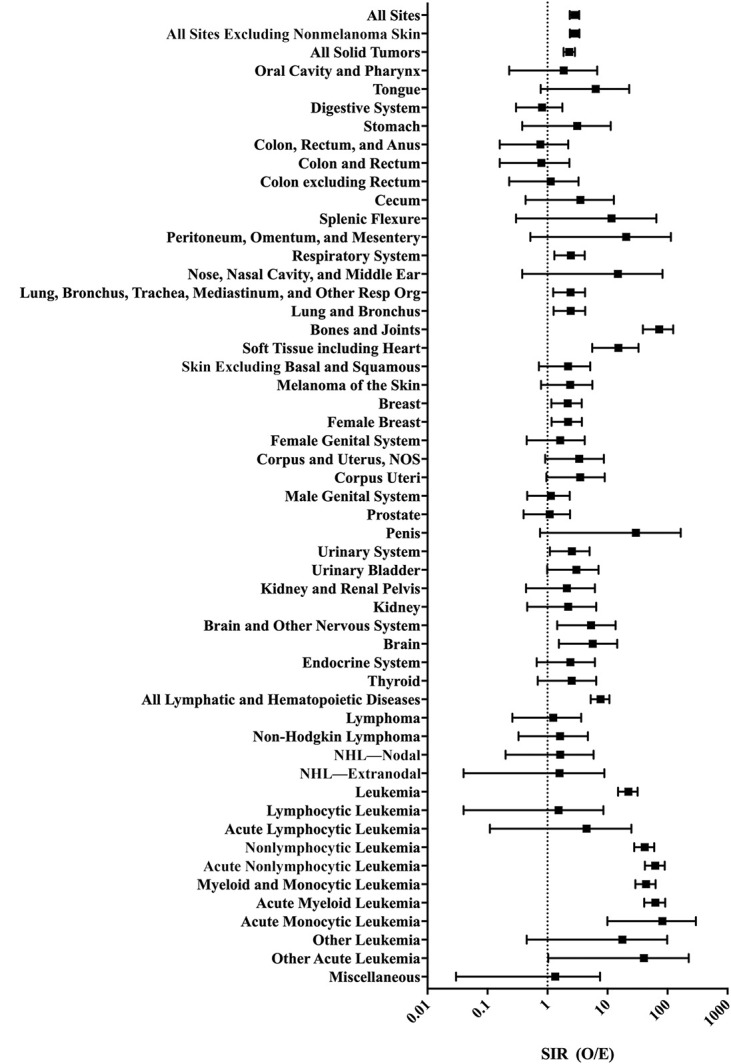
Diagram showing SIR by SPM location (95% CI). This plot shows the SIRs for SPMs across the different SPM diagnoses. SIR = standardized incidence ratio, SPM = secondprimary malignancy, CI = confidence interval, O/E = observed/expected.

With 79 SPMs identified, the incidence of SPM development had an SIR of 2.84 (CI, 2.35 to 3.39, *P* < 0.0001), Table 2. Because of overlapping anatomic site designations, 119 anatomic sites were represented across the 79 observed SPMs, 85 (71%) of these were solid tumors (SIR: 2.84, CI, 2.35 to 3.39, *P* < 0.0001) and 33 (28%) were lymphatic or hematopoietic malignancies (SIR: 7.65, CI, 5.26 to 10.74, *P* < 0.0001), Table 2 and Figure 3.

**Table 2:** Standardized Incidence Ratios for Multiple Primary Malignancies by Anatomic Site of Subsequent Primary

Site	Observed	O/E	*p* Value	95% CI		Absolute Excess Risk (Per 10,000 Persons)
				2.5	97.5	
All sites	119	2.84	0.005	2.35	3.39	44.96
All sites excluding nonmelanoma skin	119	2.85	0.004	2.36	3.41	45.06
All solid tumors	85	2.31	0.02	1.85	2.86	28.17
Oral cavity and pharynx	2	1.87	0.06	0.23	6.74	0.54
Tongue	2	6.39	<0.0001	0.77	23.09	0.98
Digestive system	6	0.81	0.42	0.30	1.77	−0.80
Stomach	2	3.13	0.002	0.38	11.30	0.79
Colon, rectum, and anus	3	0.76	0.46	0.16	2.22	−0.55
Colon and rectum	3	0.79	0.44	0.16	2.32	−0.46
Colon excluding rectum	3	1.13	0.26	0.23	3.30	0.20
Cecum	2	3.54	0.0004	0.43	12.80	0.84
Splenic flexure	1	11.71	<0.0001	0.30	65.22	0.53
Peritoneum, omentum, and mesentery	1	20.50	<0.0001	0.52	114.20	0.56
Respiratory system	13	2.45	0.01	1.30	4.18	4.49
Nose, nasal cavity, and middle ear	1	14.87	<0.0001	0.38	82.86	0.54
Lung, bronchus, trachea, mediastinum, and other resp org	12	2.43	0.02	1.25	4.24	4.12
Lung and bronchus	12	2.44	0.01	1.26	4.26	4.14
Bones and joints	13	73.07	<0.0001	38.90	124.94	7.48
Soft tissue including heart	6	15.19	<0.0001	5.58	33.07	3.27
Skin excluding basal and squamous	5	2.21	0.03	0.72	5.15	1.60
Melanoma of the skin	5	2.40	0.02	0.78	5.60	1.70
Breast	13	2.18	0.03	1.16	3.73	4.10
Female breast	13	2.20	0.03	1.17	3.75	4.13
Female genital system	4	1.63	0.10	0.45	4.19	0.91
Corpus and uterus, NOS	4	3.39	0.0007	0.92	8.69	1.65
Corpus uteri	4	3.51	0.0005	0.96	8.98	1.67
Male genital system	7	1.14	0.26	0.46	2.35	0.50
Prostate	6	1.09	0.28	0.40	2.38	0.30
Penis	1	29.81	<0.0001	0.75	166.10	0.56
Urinary system	8	2.55	0.01	1.10	5.02	2.84
Urinary bladder	5	3.04	0.002	0.99	7.09	1.96
Kidney and renal pelvis	3	2.11	0.03	0.44	6.17	0.92
Kidney	3	2.22	0.03	0.46	6.50	0.96
Brain and other nervous system	4	5.31	<0.0001	1.45	13.60	1.90
Brain	4	5.68	<0.0001	1.55	14.55	1.92
Endocrine system	4	2.41	0.02	0.66	6.18	1.37
Thyroid	4	2.54	0.01	0.69	6.50	1.42
All lymphatic and hematopoietic diseases	33	7.65	<0.0001	5.26	10.74	16.74
Lymphoma	3	1.25	0.21	0.26	3.66	0.35
Non-Hodgkin lymphoma	3	1.62	0.11	0.33	4.72	0.67
NHL—nodal	2	1.63	0.10	0.20	5.88	0.45
NHL—extranodal	1	1.59	0.11	0.04	8.86	0.22
Leukemia	30	22.28	<0.0001	15.03	31.80	16.72
Lymphocytic leukemia	1	1.53	0.13	0.04	8.55	0.20
Acute lymphocytic leukemia	1	4.49	<0.0001	0.11	24.99	0.45
Nonlymphocytic leukemia	29	41.75	<0.0001	27.96	59.95	16.52
Acute nonlymphocytic leukemia	29	62.60	<0.0001	41.92	89.90	16.66
Myeloid and monocytic leukemia	28	43.86	<0.0001	29.15	63.40	15.97
Acute myeloid leukemia	26	62.76	<0.0001	41.00	91.96	14.93
Acute monocytic leukemia	2	82.12	<0.0001	9.95	296.66	1.15
Other leukemia	1	17.75	<0.0001	0.45	98.89	0.55
Other acute leukemia	1	40.55	<0.0001	1.03	225.91	0.57
Miscellaneous	1	1.35	0.18	0.03	7.54	0.15

SIR = standardized incidence ratio, CI = 95% confidence interval, AER = absolute excess risk per 10,000 persons, Sig = significance. *P < 0.05, **P < 0.0009.

After corrections for multiple comparisons, the trends for overall SPMs in lymphatic and hematopoietic diseases remained significant, with leukemia being the significant SPM with the highest effect size (SIR: 22.28, CI, 15.03 to 31.80, *P* < 0.0001), Table 2 and Figure 3. After correcting for multiple comparisons, significant solid tumor SPM sites were bones and joints (SIR: 73.07, CI, 38.90 to 124.94, *P* < 0.0001) and soft tissues including the heart (SIR: 15.19, CI, 5.58 to 33.07, *P* < 0.0001), Table 2 and Figure 3.

When assessing AER, SPMs were significantly more likely to arise in several locations in osteosarcoma survivors (all sites excluding nonmelanoma skin, AER: 44.96), particularly tumors of the bone and joint (AER: 7.48), oral cavity and pharynx (AER: 28.17), soft tissue including heart (AER: 7.48), skin excluding basal and squamous (AER: 3.27), lymphoma (AER: 16.74), lymphocytic leukemia (AER: 16.72), acute nonlymphocytic leukemia (AER: 16.52), myeloid and monocytic leukemia (AER: 16.66), acute myeloid leukemia (AER: 15.97), acute monocytic leukemia (AER: 14.93), and other leukemia (AER: 1.15), Table 2.

## Discussion

This study of the SEER database examining and updating the incidence and characteristics of SPMs in osteosarcoma survivors found that the cumulative incidence of SPMs was significantly higher than expected for the population based on baseline population rates. In particular, standardized absolute and relative incidence of malignancies in the bones and joints, soft tissue including the heart, and lymphoid and hematopoietic malignancies were significantly higher than expected.

Second primary malignancies are a devastating long-term sequela for many survivors of cancers. According to Soerjomataram and Coebergh,^17^ patients with cancer have a 20% higher risk of developing a new primary cancer compared with the general population. Many causal factors are believed to contribute to the development of SPMs. Radiation, for example, has long been observed to induce secondary malignancies at the site of exposure.^18-20^ Chemotherapy, known for its leukemogenic properties, and its adjuncts, such as high-dose methotrexate, doxorubicin, and cisplatin, have also been linked to SPM development.^21-23^ A family history of Li-Fraumeni syndrome, an autosomal dominant genetic disorder associated with a variety of malignancies including primary osteosarcoma, can also contribute to the risk of SPM.^24^

In many ways an update to Lee et al.’s initial SEER study examining SMNs in osteosarcoma survivors, this study represents the most recent estimates and characterization of SPMs in osteosarcoma survivors using the largest data source to date. The current study replicated Lee et al.’s finding of the cumulative incidence of SPM’s in this population, here finding an estimate of 2.3%, close to Lee et al.’s estimate of a 2.1% 10-year cumulative incidence. However, the results of this study differ from those of Lee et al. in several ways, indicating the changing landscape of care and outcomes for this population. This study sample is more recent (including data up to 2015 vs. 2010) and includes patients diagnosed with osteosarcoma in all age groups. Based on this, this study found a lower SIR of leukemia (22.3 vs. 34.8) and a higher SIR of tumors of the bones and joints (73.1 vs. 11.0). Finally, the cumulative SIR in the study of Lee et al. from 1986 to 2010 was 4.7 (CI, 3.3 to 6.4), while in this study we found a cumulative SIR from 2000 to 2015 to be 2.84 (CI, 2.35 to 3.39), potentially elucidating a dramatic decrease in cumulative incidence of SPMs in recent years.

Before the findings of Lee et al, several small retrospective studies had investigated the incidence of SPMs or SMNs. In a retrospective chart review, 509 consecutive patients treated at Memorial Sloan-Kettering Cancer Center between 1973 and 2000 reported a 10-year incidence of 3.1% where the most common site of SPM was central nervous system, with 1 case of fibrosarcoma in this cohort.^10^ Bacci et al. and Longhi et al., in their studies on Italian patient cohorts, reported 10-year cumulative incidence rates of 4.6% and 4.9% for SPMs, respectively.^11,13^ The Childhood Cancer Survivor Study has been analyzed by many groups, resulting in rates between 0.5% and 1.0% at 10 years.^25,26^

## Limitations

Although wide-ranging and informative, this study has several limitations. The SEER database does not include 72% of the US population and does not account for patients who may have moved out of a SEER area, possibly leading to an underestimation of the risk of SPMs. SEER does not collect data regarding factors that may play a role in the development of an SPM such as smoking, alcohol use, exposure to environmental factors, comorbidities, or family history. In addition, lack of specific data on tumor location in the body, histologic subtype treatments, outcomes (e.g., survival, complications), and healthcare cost and utilization data further limited our analyses. Furthermore, our study has limitations associated with retrospective analyses, including a lack of causal explanatory power. A possible explanation for the observed rate of SPMs in patients with osteosarcoma that is reported here and elsewhere is detection bias because of increased medical scrutiny of this population.

## Conclusion

This study demonstrated that osteosarcoma survivors in the United States are at an increased risk for developing a range of SPMs, characterizes those SPMs by age of development and tumor location, and updates prior estimates. As the treatments for osteosarcoma improve leading to increased survival rates, the results of our study demonstrate the importance of routine monitoring of patients who have had osteosarcoma. Further studies should investigate the association between chemotherapeutics, radiation therapy, and SPMs in osteosarcoma survivors.

## References

[1] Mirabello L Troisi RJ Savage SA: International osteosarcoma incidence patterns in children and adolescents, middle ages and elderly persons. Int J Cancer 2009;125:229-234. 19330840 10.1002/ijc.24320 PMC3048853

[2] Bielack SS Kempf-Bielack B Delling G : Prognostic factors in high-grade osteosarcoma of the extremities or trunk: An analysis of 1, 702 patients treated on neoadjuvant cooperative osteosarcoma study group protocols. J Clin Oncol 2002;20:776-790. 11821461 10.1200/JCO.2002.20.3.776

[3] Anfinsen KP Devesa SS Bray F : Age-period-cohort analysis of primary bone cancer incidence rates in the United States (1976-2005). Cancer Epidemiol Biomarkers Prev 2011;20:1770-1777. 21724855 10.1158/1055-9965.EPI-11-0136

[4] Mirabello L Troisi RJ Savage SA: Osteosarcoma incidence and survival rates from 1973 to 2004: Data from the surveillance, Epidemiology, and End results program. Cancer 2009;115:1531-1543. 19197972 10.1002/cncr.24121 PMC2813207

[5] Shapiro CL Recht A: Side effects of adjuvant treatment of breast cancer. New Engl J Med 2001;344:1997-2008. 11430330 10.1056/NEJM200106283442607

[6] Schaapveld M Aleman BM van Eggermond AM : Second cancer risk up to 40 Years after treatment for hodgkin's lymphoma. New Engl J Med 2015;373:2499-2511. 26699166 10.1056/NEJMoa1505949

[7] Landgren O Thomas A Mailankody S: Myeloma and second primary cancers. New Engl J Med 2011;365:2241-2242. 22150057 10.1056/NEJMc1111010 PMC7213755

[8] Victoria K Freedman IG: De novo myelodysplastic syndrome and subsequent diagnosis of primary solid tumors: Evidence from the national cancer Institute 2001-2011. Anticancer Res 2018;38:5819-5823. 30275205 10.21873/anticanres.12922

[9] Lee JS DuBois SG Boscardin WJ Wustrack RL Goldsby RE: Secondary malignant neoplasms among children, adolescents, and young adults with osteosarcoma. Cancer 2014;120:3987-3993. 25116228 10.1002/cncr.28936

[10] Aung L Gorlick RG Shi W : Second malignant neoplasms in long-term survivors of osteosarcoma: Memorial sloan-kettering cancer center experience. Cancer 2002;95:1728-1734. 12365021 10.1002/cncr.10861

[11] Bacci G Ferrari C Longhi A : Second malignant neoplasm in patients with osteosarcoma of the extremities treated with adjuvant and neoadjuvant chemotherapy. J Pediatr Hematol Oncol 2006;28:774-780. 17164644 10.1097/01.mph.0000243664.02174.73

[12] Pratt CBMW Meyer WH Luo X : Second malignant neoplasms occurring in survivors of osteosarcoma. Cancer 1997;80:960-965. 9307198 10.1002/(sici)1097-0142(19970901)80:5<960::aid-cncr19>3.0.co;2-u

[13] Longhi A Ferrari S Tamburini A : Late effects of chemotherapy and radiotherapy in osteosarcoma and ewing sarcoma patients: The Italian sarcoma group experience. Cancer 2012;118:5050-5059. 22415578 10.1002/cncr.27493

[14] Institute NC. Follow-up process. 2019. https://training.seer.cancer.gov/followup/process/. Accessed August 1, 2021.

[15] Institute NC. Requirements of CoC/governing agencies. 2019. https://training.seer.cancer.gov/followup/intro/requirements.html. Accessed August 1, 2021.

[16] Altman DG Bland JM: How to obtain the P value from a confidence interval. BMJ 2011;343:d2304. 22803193 10.1136/bmj.d2304

[17] Soerjomataram I Coebergh JW: Epidemiology of multiple primary cancers. Methods Mol Biol (Clifton, NJ) 2009;471:85-105. 10.1007/978-1-59745-416-2_5 19109776

[18] Bhatia S Robison LL Oberlin O : Breast cancer and other second neoplasms after childhood Hodgkin's disease. New Engl J Med 1996;334:745-751. 8592547 10.1056/NEJM199603213341201

[19] Hutchison GB: Late neoplastic changes following medical irradiation. Cancer 1976;37:1102-1107. 766955 10.1002/1097-0142(197602)37:2+<1102::aid-cncr2820370819>3.0.co;2-q

[20] Li FP: Second malignant tumors after cancer in childhood. Cancer 1977;40:1899-1902. 907992 10.1002/1097-0142(197710)40:4+<1899::aid-cncr2820400821>3.0.co;2-u

[21] Orazi A Sozzi C Delia D Morandi F Rottoli L Cattoretti G: Acute monoblastic leukemia as a second malignancy following chemotherapy for osteogenic sarcoma: A case report. Pediatr Hematol Oncol 1988;5:39-46. 3152950 10.3109/08880018809031250

[22] Pappo A Schneider NR Sanders JM Buchanan GR: Secondary myelodysplastic syndrome complicating therapy for osteogenic sarcoma. Cancer 1991;68:1373-1375. 1714791 10.1002/1097-0142(19910915)68:6<1373::aid-cncr2820680631>3.0.co;2-s

[23] Whang-Peng J Young RC Lee EC Longo DL Schechter GP DeVita VJ: Cytogenetic studies in patients with secondary leukemia/dysmyelopoietic syndrome after different treatment modalities. Blood 1988;71:403-414. 3337904

[24] McIntyre JF Smith-Sorensen B Friend SH : Germline mutations of the p53 tumor suppressor gene in children with osteosarcoma. J Clin Oncol 1994;12:925-930. 8164043 10.1200/JCO.1994.12.5.925

[25] Hospital SJCsR. The childhood cancer survivor study: Baseline data. 2018 stjude.org/ccss. Accessed August 1, 2021.

[26] Neglia JP Friedman DL Yasui Y : Second malignant neoplasms in five-year survivors of childhood cancer: Childhood cancer survivor study. JNCI J Natl Cancer Inst 2001;93:618-629. 11309438 10.1093/jnci/93.8.618

